# Supplementary material for: Novel genes arise from genomic deletions across the bacterial tree of life
Source: bioRxiv. 2026 Jan 5:2026.01.05.697752. Preprint. [Version 1] doi: 10.64898/2026.01.05.697752 (PMC12803068; doi:10.64898/2026.01.05.697752)
Supplement: Supplement 3 [file NIHPP2026.01.05.697752v1-supplement-3.pdf]

bioRxiv preprint doi: https://doi.org/10.64898/2026.01.05.697752; this version posted January 5, 2026. The copyright holder for this preprint (which was not certified by peer review) is the author/funder, who has granted bioRxiv a license to display the preprint in perpetuity. It is made available under aCC-BY 4.0 International license.

SUPPLEMENTARY NOTE(S)

Estimation of yjcO-lysU/deletion selection coefficient

For a haploid population in which a novel beneficial allele has fitness 1 + s, and the wildtype has a fitness of 1, the allele-frequency dynamics are given by:

$$p_{t+1} = \frac{p_t(1+s)}{1+sp_t}$$

Where p_t is the frequency of the novel allele at generation t. This can be approximated to the differential equation, a classical result from Kimura:[69]

$$\frac{dp}{dt} = sp(1-p)$$

Integrating over this equation and solving for t gives:[69]

$$t \approx \frac{1}{s} ln\left(\frac{p_1(1-p_0)}{p_0(1-p_1)}\right)$$

Conditioning on the allele fixing, we can set $p_0 = \frac{1}{N_e}$ and $p_1 = 1 - \frac{1}{N_e}$. For large effective populations, this yields the commonly used approximation:

$$t \approx \frac{2}{s} ln(N_e)$$

Solving for s gives:

$$s \approx \frac{2ln(N_e)}{t}$$

From Good et al.,[39] we estimate $N_e = 10^7$, from our metagenomic analysis, we estimate $t = 500$:

$$s \approx \frac{2ln(10^7)}{500} \approx 0.065$$

Yielding the estimated selection coefficient of 6.5% for the *yjcO-lysU* fusion/deletion.

bioRxiv preprint doi: https://doi.org/10.64898/2026.01.05.697752; this version posted January 5, 2026. The copyright holder for this preprint (which was not certified by peer review) is the author/funder, who has granted bioRxiv a license to display the preprint in perpetuity. It is made available under aCC-BY 4.0 International license.

SUPPLEMENTAL INFORMATION

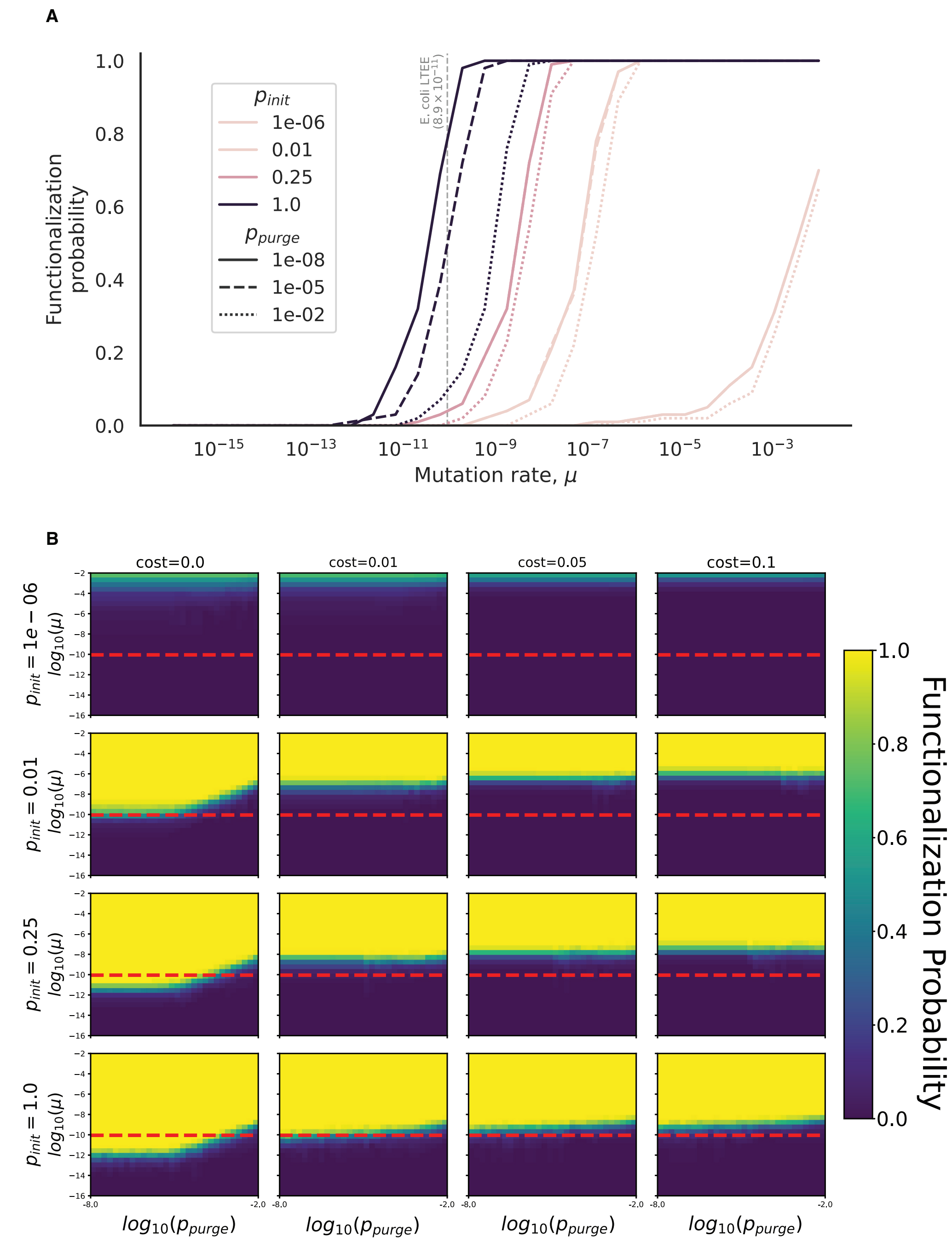

Supplementary Figure 1. Forward simulations quantify the hitch-hiking advantage
(**A**) Probability that at least one lineage functionalizes as a function of the mutation rate μ; colors denote four starting frequencies of the nascent fusion (p_{init}) and line styles three purge probabilities (p_{purge}). The dashed vertical line marks the LTEE point-mutation rate. All curves are shown for a fusion gene with a pre-functionalized fitness cost of 0.01.

(**B**) Heat-maps show the same probability across grids of p_{purge} (x-axis, $\log_{10}$ scale) and fitness cost of the unfixed fusion (c, four columns) for the four p_{init} values (rows); the color of the cell indicates the proportion of times the fusion functionalized before being purged. The dashed red line denotes the LTEE point-mutation rate. Each cell summarizes 100 Wright–Fisher runs of 10^6 haploids.

bioRxiv preprint doi: https://doi.org/10.64898/2026.01.05.697752; this version posted January 5, 2026. The copyright holder for this preprint (which was not certified by peer review) is the author/funder, who has granted bioRxiv a license to display the preprint in perpetuity. It is made available under aCC-BY 4.0 International license.

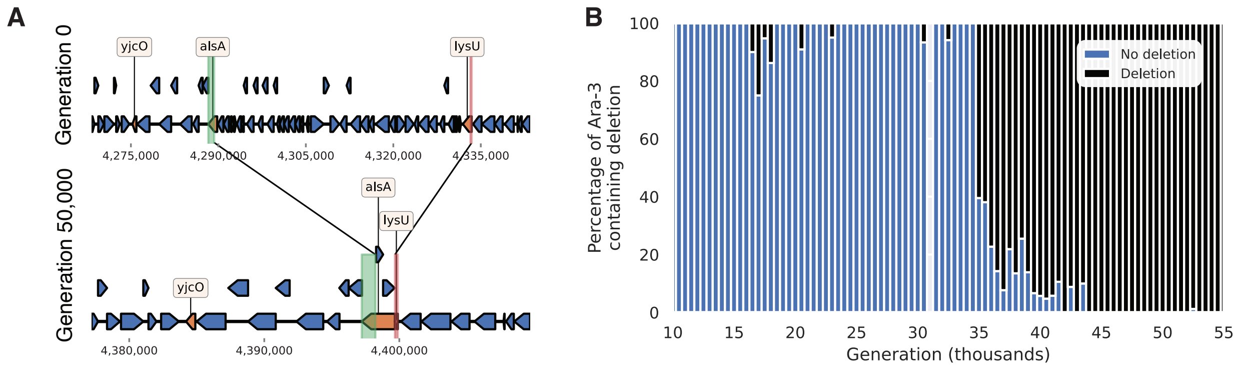

Supplementary Figure 2. A convergent 43.4 kb deletion sweeps at the same locus in Ara-3.
(**A**) Schematic of the deletion in Ara-3, orange genes highlight the resulting prior genes involved in the deletion. Green and red bars correspond to BLAST alignments to this region.

(**B**) Metagenomic sequencing results showing the fraction of reads supporting the deletion or not.

bioRxiv preprint doi: https://doi.org/10.64898/2026.01.05.697752; this version posted January 5, 2026. The copyright holder for this preprint (which was not certified by peer review) is the author/funder, who has granted bioRxiv a license to display the preprint in perpetuity. It is made available under aCC-BY 4.0 International license.

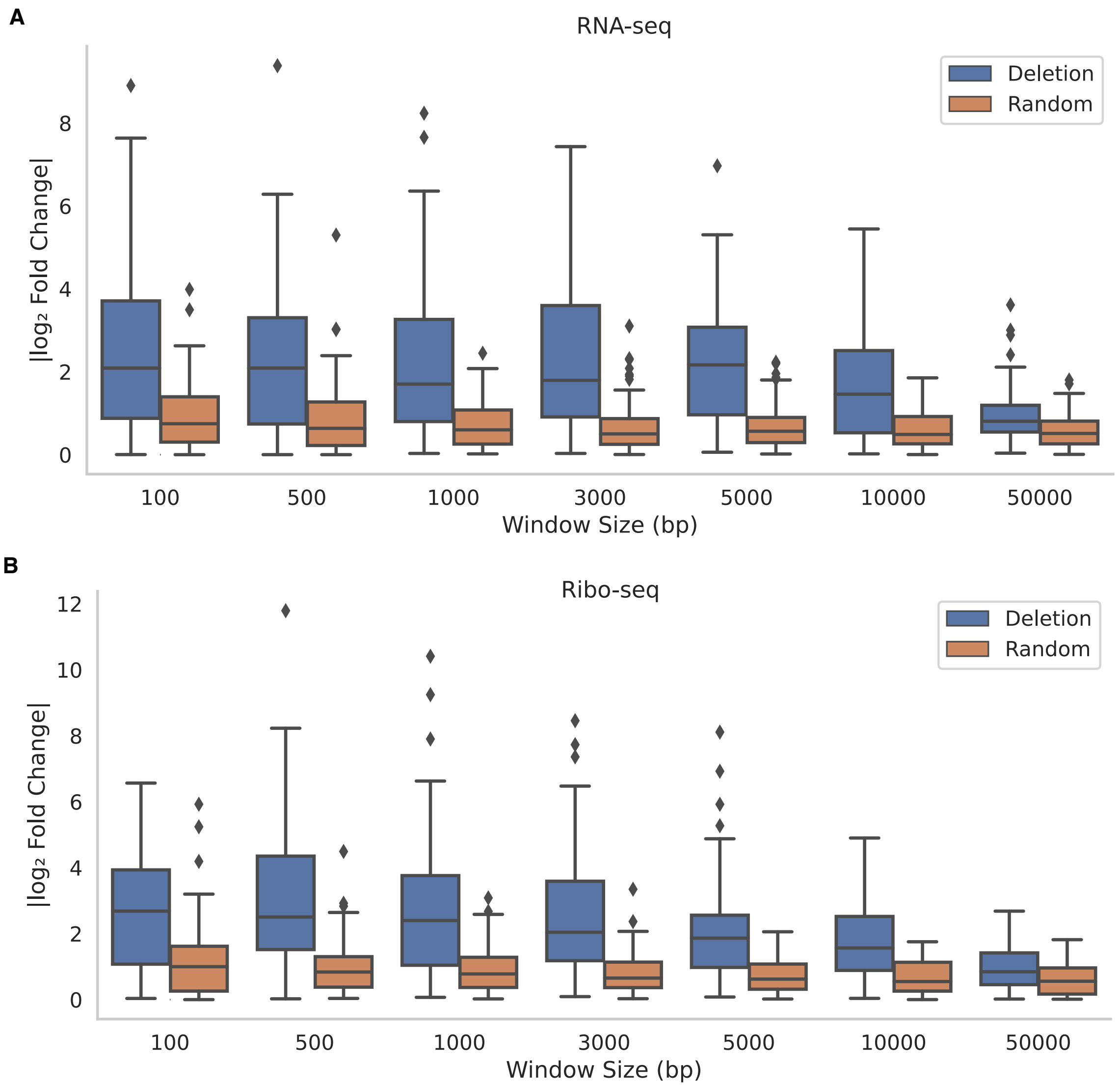

Supplementary Figure 3. Large LTEE deletions are associated with significant changes to local transcription and translation.

(**A**) RNA-seq $\log_2$ fold changes for windows of varying size flanking ≥1 kb deletions (blue) and for randomly sampled windows (orange). Fold changes are calculated between the ancestral strain and the evolved population at generation 50,000. Data downloaded from Favate et al. 2022.[40]

(**B**) As in (A) but analyzing Ribo-seq data.

bioRxiv preprint doi: https://doi.org/10.64898/2026.01.05.697752; this version posted January 5, 2026. The copyright holder for this preprint (which was not certified by peer review) is the author/funder, who has granted bioRxiv a license to display the preprint in perpetuity. It is made available under aCC-BY 4.0 International license.

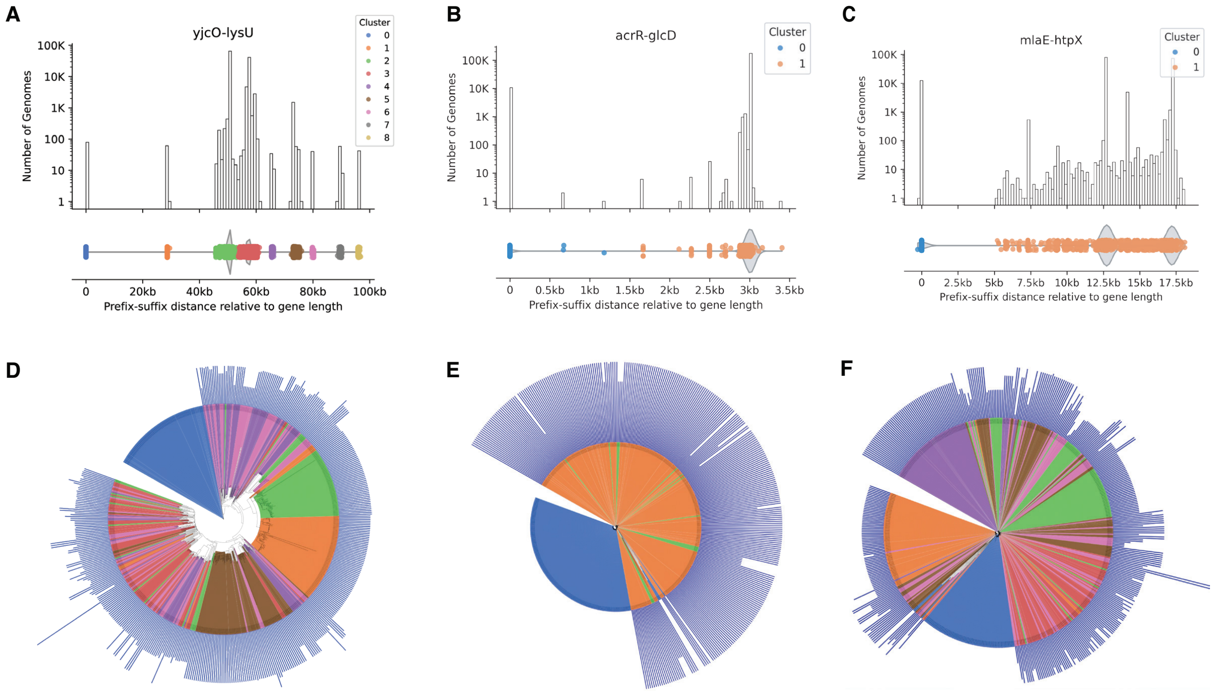

Supplementary Figure 4. The prefix-suffix approach captures previously identified deletion-born fusions and reveals additional structural variation.

(**A**) Top: Log-scaled histogram of prefix-suffix distances relative to the length of the original *yjcO-lysU* fusion. Bottom: underlying point distribution for histogram. Every point represents a single genome in the ATB dataset; colors correspond to DBSCAN-derived clusters.

(**B**), (**C**) are the same as in (A) but with *acrR-glcD* and *mlaE-htpX* respectively.

(**D**) Distance-metric based phylogeny of 300 randomly sampled genomes with equal genomes sampled across the number of clusters identified. Clades are colored by cluster membership, and blue vertical bars off leaves represent the distance between the prefix-suffix found in that genome relative to the *yjcO-lysU* gene.

(**E**), (**F**) are the same as in (D) but with *acrR-glcD* and *mlaE-htpX* respectively.

bioRxiv preprint doi: https://doi.org/10.64898/2026.01.05.697752; this version posted January 5, 2026. The copyright holder for this preprint (which was not certified by peer review) is the author/funder, who has granted bioRxiv a license to display the preprint in perpetuity. It is made available under aCC-BY 4.0 International license.

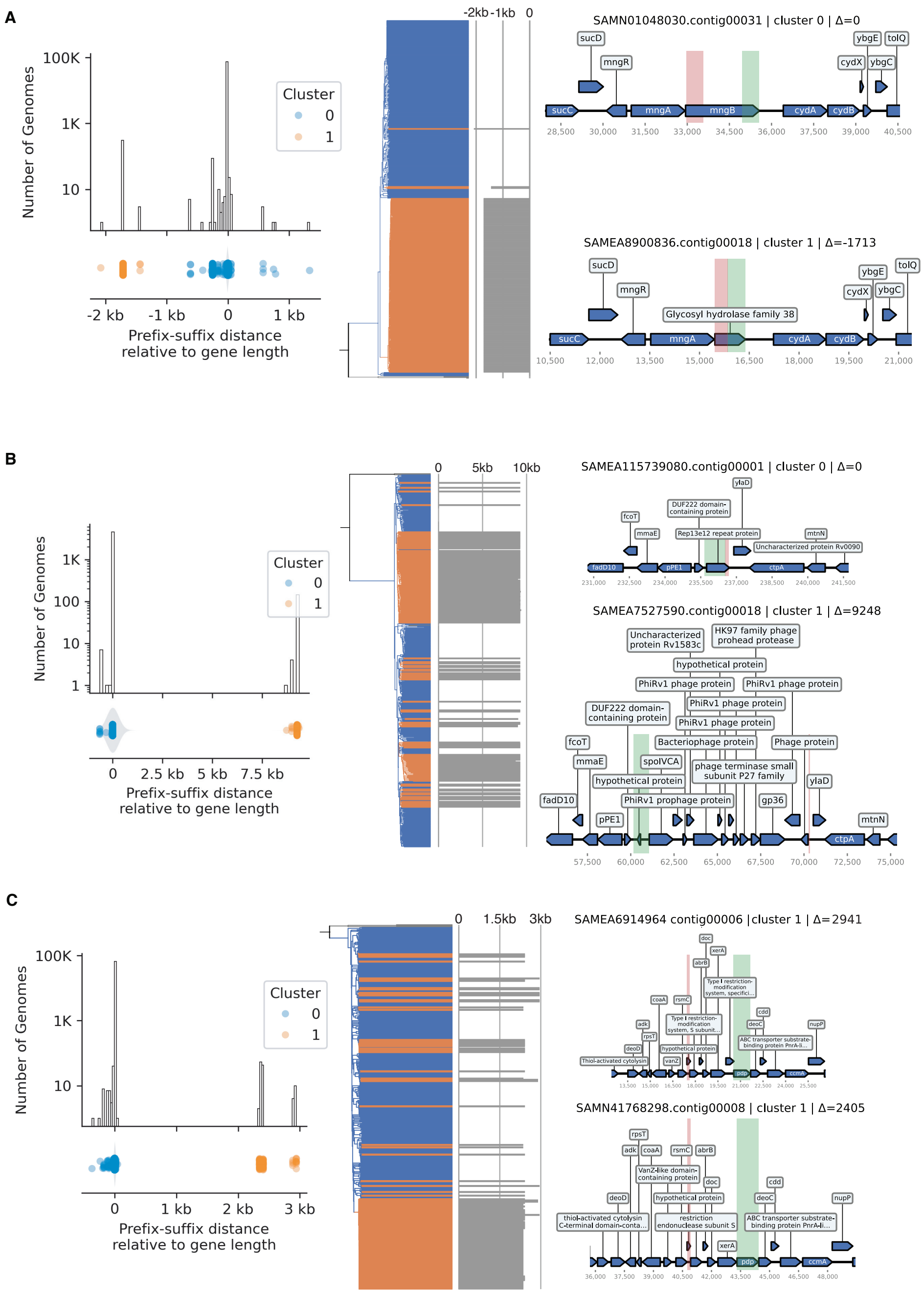

Supplementary Figure 5. Prefix-suffix approach captures diverse structural variation beyond deletion-born fusions.

Each row shows: prefix-suffix distance distribution (left), rooted phylogeny of 300 sampled genomes (middle), and representative genomic contexts (right).

(**A**) Internal deletion in *mngB* (*E. coli* K12).

(**B**) Repeat prophage insertions in *rep13e12* (*M. tuberculosis* H37Rv).

(**C**) Variable gene cargo disrupting *pdp* (*S. pneumoniae* TIGR4); the two Cluster 1 representatives differ by ~500 bp due to the presence/absence of a Type I restriction system protein downstream of *xerA*.

bioRxiv preprint doi: https://doi.org/10.64898/2026.01.05.697752; this version posted January 5, 2026. The copyright holder for this preprint (which was not certified by peer review) is the author/funder, who has granted bioRxiv a license to display the preprint in perpetuity. It is made available under aCC-BY 4.0 International license.

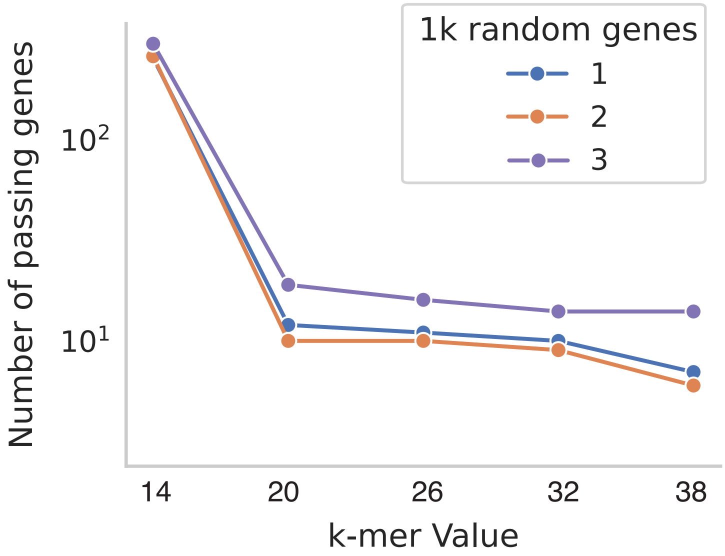

Supplementary Figure 6. Prefix-suffix approach is robust to values of k ≥ 20.
Number of genes with multimodal prefix-suffix distances detected across three random samples of 1,000 RefSeq genes at varying k-mer lengths.

bioRxiv preprint doi: https://doi.org/10.64898/2026.01.05.697752; this version posted January 5, 2026. The copyright holder for this preprint (which was not certified by peer review) is the author/funder, who has granted bioRxiv a license to display the preprint in perpetuity. It is made available under aCC-BY 4.0 International license.

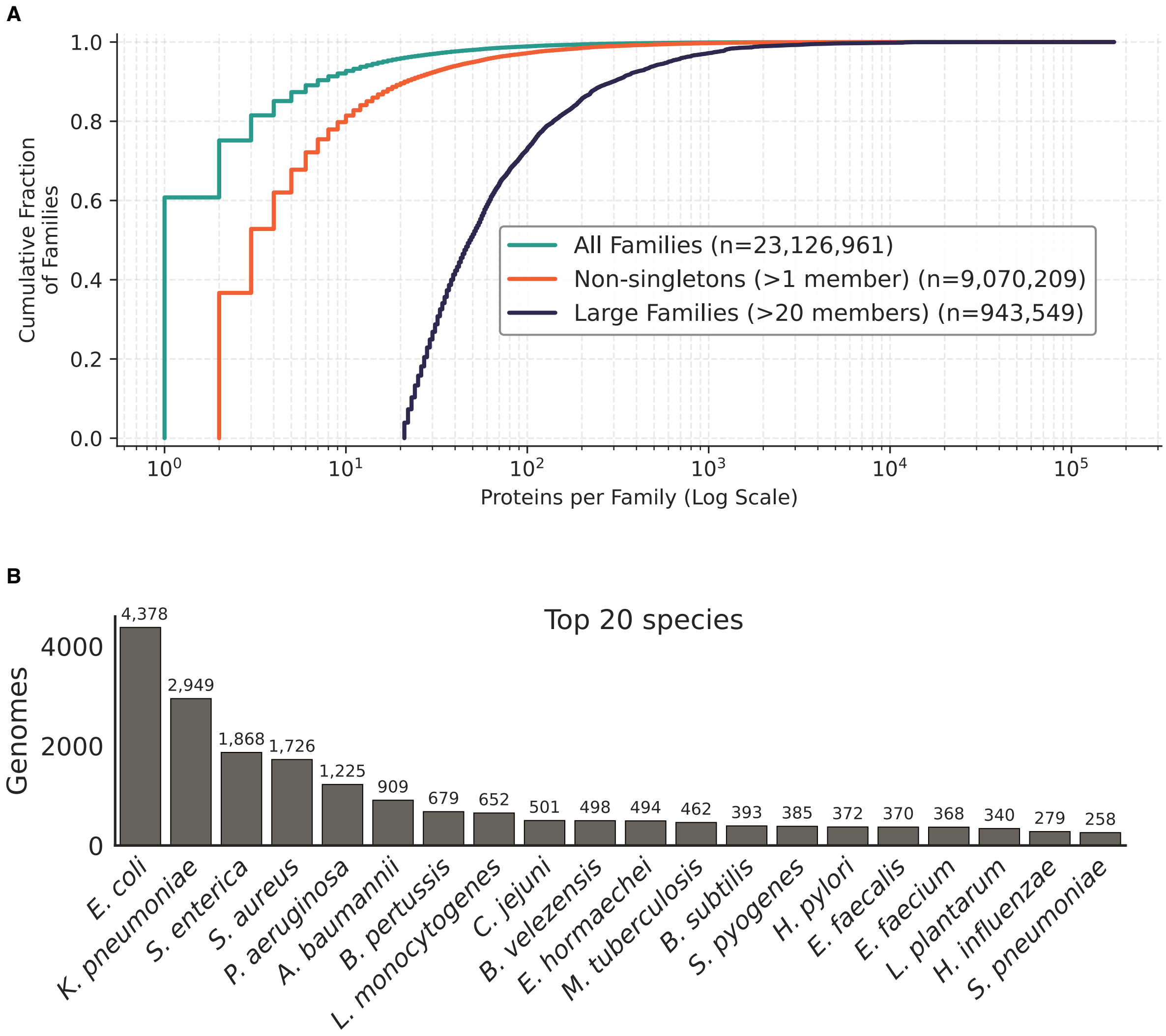

Supplementary Figure 7. Representation of both protein families and bacterial species are highly skewed in RefSeq complete genomes.

(**A**) Cumulative distribution function of the number of protein members per family. Most represented proteins are singletons

(**B**) Top 20 species represented in the 54,630 complete genomes proteins were pulled from. Count of each is displayed above each bar.
